# Barrett’s esophagus network analysis revealed that arginine, alanine, aspartate, glutamate, valine, leucine and isoleucine can be biomarkers 

**Published:** 2018

**Authors:** Mohammad Reza Zali, Mohammad-Mehdi Zadeh-Esmaeel, Majid Rezaei Tavirani, Sina Rezaei Tavirani, Mohsen Norouzinia, Mostafa Rezaei-Tavirani

**Affiliations:** 1 *Gastroenterology and Liver Diseases Research Center, Research Institute for Gastroenterology and Liver Diseases, Shahid Beheshti University of Medical Sciences, Tehran, Iran *; 2 *Skin Research Center, Shahid Beheshti University of Medical Sciences, Tehran, Iran*; 3 *Faculty of Medicine, Iran University of Medical Sciences, Tehran, Iran*; 4 *Proteomics Research Center, Shahid Beheshti University of Medical Sciences, Tehran, Iran*; 5 *Proteomics Research Center, Faculty of Paramedical Sciences, Shahid Beheshti University of Medical Sciences, Tehran, Iran *

**Keywords:** Biomarker, Barrett’s esophagus, Network

## Abstract

**Aim:**

Identification of crucial genes and possible biomarkers which are involved in Barrett’s esophagus (BE) disease was aim of this study.

**Background:**

BE is diagnosed by endoscopy and biopsy and is characterized by esophageal columnar metaplastic epithelium. BE can convert into dysplasia that finally results cancer condition.

**Methods:**

Gene expression profiles of BE and normal gastric cardia which are characterized by GSE34619 and GPL6244 platform (1) were retrieved from gene expression omnibus (GEO). The significant differentially expressed genes (DEGs) were analyzed via protein-protein interaction network (PPI) analysis. The nodes of network were enriched via gene ontology (GO) to find biological terms. Action map of network elements was provided.

**Results:**

Among 250 top DEGs, 100 ones were included in PPI network and KIT, CFTR, IMPDH2, MYB, FLT1, ATP4A, and CPS1 were recognized as prominent genes related to BE. Seven amino acids including arginine, alanine, aspartate, glutamate, valine, leucine and isoleucine which are related to BE were highlighted.

**Conclusion:**

In conclusion five central DEGs; KIT, CFTR, IMPDH2, MYB, and FLT1 were proposed as possible biomarkers for BE. However, validation and more experimental information is require to finalize the findings.

## Introduction

 Barrett’s esophagus (BE) is considered as a precancerous condition which is characterized by esophageal columnar metaplastic epithelium. BE progressive converts into dysplasia. There is evidence that dysplasia can lead to cancer condition. Two aggressive methods including endoscopy and biopsy are the main diagnostic tools for BE (2, 3). There are serious attempts to find and replace non-aggressive diagnostic methods instead these types of destructive tools. The molecular based methods are attractive ones due to easy access to biofluids (4, 5). High throughout methods such as proteomics and Genomics can provide large amounts of data about diseases, therefore are attracted attention of clinical researchers to find new effective and valid biomarkers for early detection and follow up of disorders (6, 7). In these approaches, huge numbers of differentially expressed genes (DEGs) and proteins attributes to a certain disorder. Different amounts of fold change differentiate the identified biomolecules (8). Bioinformatics and system biology facility helps to analysis complex systems to find important biomolecules among the other ones. Screening and analysis of DEGs via protein-protein interaction (PPI) network is a useful method to find the prominent gens which play critical role in disorder. Central parameters such as degree, betweenness centrality, and closeness centrality are used to find such crucial genes (9-11). Gene ontology is the other activity that is applied to determine functional features of query genes. Molecular function, biological processes, cellular component, and biochemical pathways related to the investigated genes identify via gene ontology analysis (12, 13). In this study significant DEGs of BE tissue relative to normal gastric cardia samples are analyzed by PPI network method and enriched via GO to be screened and finding critical genes which are involved in onset and progression of BE in patients. It may be conduct to introduce some diagnostic metabolites for disorder. 

## Methods

Gene expression data series GSE34619, GPL6244 platform (14) were obtain from GEO database. 10 differential gene expression profiles of BE (GSM851983-92) and 10 normal gastric cardia (1) samples (GSM852001-10) were matched via boxplot analysis. The gene expression profiles were analyzed by GEO2R and the top 250 significant DEGs were selected. P-value less than 0.05, 2≤ fold change (FC) ≤ 0.5 were considered and the uncharacterized individuals were excluded. 

The determined DEGs were included in PPI network analysis via STRING database (15) and Cytoscape software (16). The main connected component was identified as network and was analyzed by Network analyzer plugin of Cytoscape. The top 10 central nodes were identified separately based on degree, betweenness, and closeness centralities. The common nodes among central nodes were introduced as critical genes.

Action map including expression, activation, and inhibition actions was provided for elements of the main connected component via CluePedia (17). The biochemical pathways relative to the all nodes of the main connected component were identified from KEGG (18) by clueGO (19).

For more investigation the query DEGs were grouped based on logarithm of fold change and the most deregulated groups were analyzed. 

## Results

**Figure 1:**
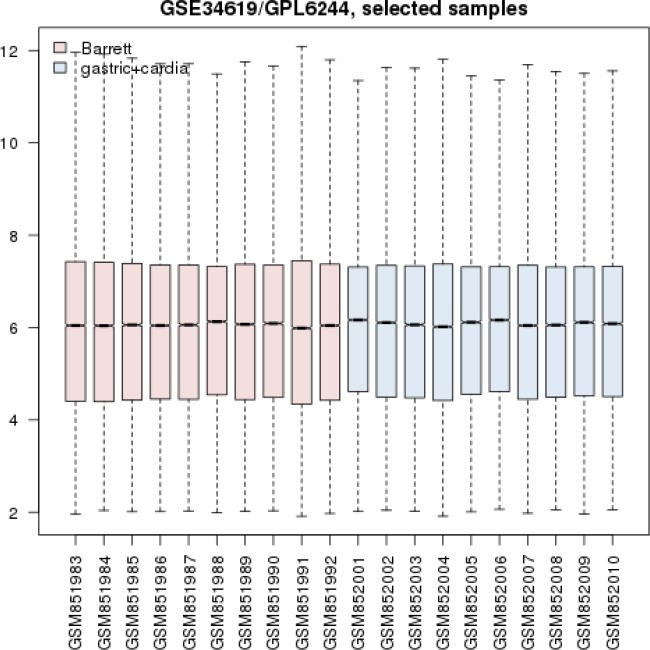
Box plot representation of gene expression profiles of BE and gastric cardia samples

Statistical matching of Gene profiles of 10 BE samples and 10 individuals normal cardia is presented in the figure 1. 

**Figure 2:**
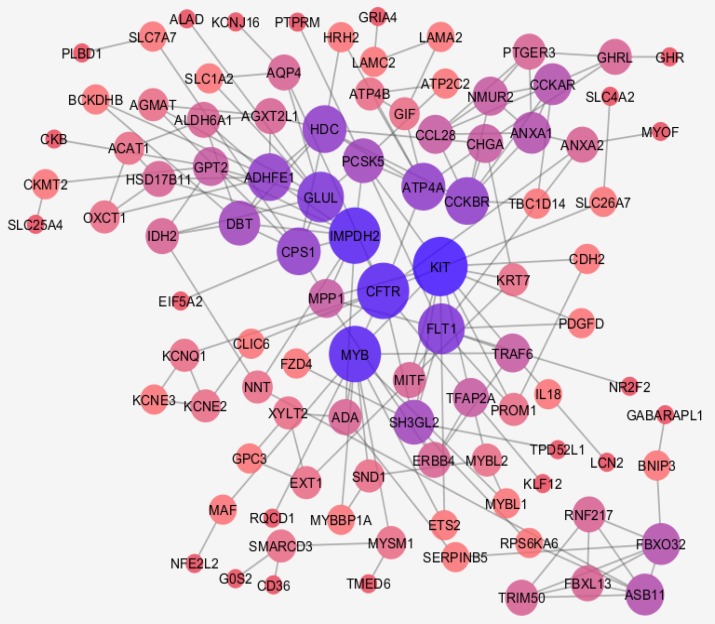
Main connected component of PPI network of BE relative to gastric cardia

**Figure 3:**
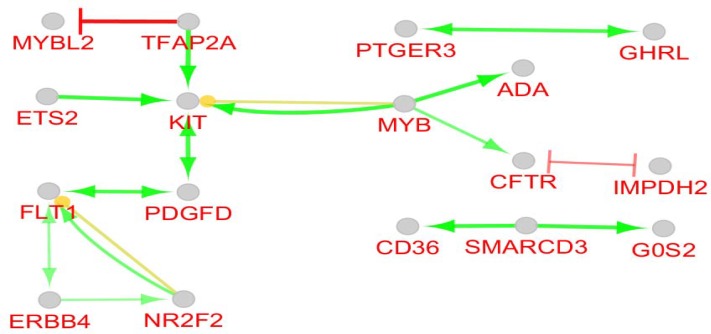
Action map of 100 nodes of main connected component is illustrated. Yellow, Green, and red colored arrow refer to expression, activation, and inhibition actions, respectively

Among 250 top changed expressed DEGs 231 ones were identified which had fold change more than 2 and less than 0.5 that were selected to further analysis. Numbers of 206 DEGs among 231 selected genes were characterized which included to construct network. As it is shown in the figure 2, the network including 95 isolated nodes, 4 paired genes, 1 triple component, and a main connected component (contain 100 nodes and 178 edges) was built. Numbers of 7 central nodes based of degree value, betweenness centrality, and closeness centrality values were identified (see table 1). Action map of 100 nodes of the main connected components including expression, activation, and inhibition actions were created, which is illustrated in the figure 3. 

**Figure 4:**
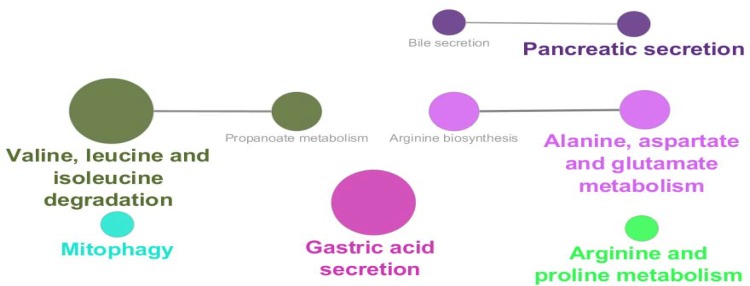
Biochemical pathways relative to 100 nodes of main connected component are shown. The pathways are retrieved from KEGG_20.11.2017

**Figure 5:**
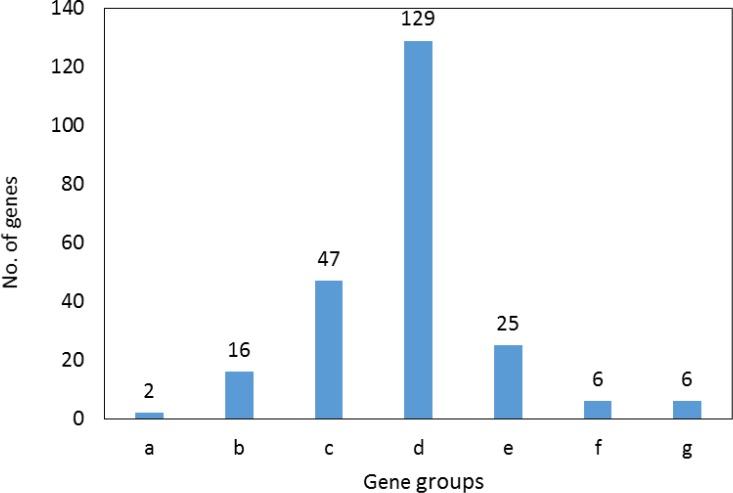
Distribution of 231 DEGs based on logarithm of fold change. Range of fold change is (-7) to 9. Group with range of logarithm fold change (-1) to 1 which refer to unselected DEGs excluded

Numbers of 9 biochemical pathways related to the elements of the main connected component which are clustered in 6 and their statistical properties groups are presented in figure 4 and table 2. Distribution of 231 DEGs based on fold change value is shown in figure 5. Minimum and maximum logarithms of fold changes were considered as -7 to 9, respectively and the samples were distributed in 7 gropes. Logarithms amounts between -1 and 1 were not selected. Elements of first most down-regulated group and first and second top up-regulated groups of genes were selected and tabulated in the table 3.

## Discussion

Network analysis can provide precise information about diseases. In this regard comparison of BE gene expression profile and esophageal cancer is studied via artificial neural network analysis (20). Here gene expression profiles of BE and Normal gastric cardi tissue are compared via PPI network analysis. As it is shown in the figure 1, gene expression profiles of BE and gastric cardia are comparable. Data are median centric and expression patterns are matched. Among 231 DEGs 100 ones are interacted in interactome (see figure 2). 

**Table 1:** Central nodes of Main connected component of PPI network of BE relative to gastric cardia,. BC and CC refer to betweenness centrality and closeness centrality, respectively. The common genes between top nodes based on Degree, BC, and CC are shown in red color

R	display name	description	BC	CC	Degree
1	KIT	v-kit Hardy-Zuckerman 4 feline sarcoma viral oncogene homolog	0.12969	0.34375	12
2	CFTR	cystic fibrosis transmembrane conductance regulator (ATP-binding cassette sub-family C, member 7)	0.332112	0.385214	11
3	IMPDH2	IMP (inosine 5'-monophosphate) dehydrogenase 2	0.269152	0.366667	11
4	MYB	v-myb myeloblastosis viral oncogene homolog (avian)	0.250107	0.346154	11
5	FLT1	fms-related tyrosine kinase 1 (vascular endothelial growth factor/vascular permeability factor receptor)	0.076052	0.326733	9
6	GLUL	glutamate-ammonia ligase	0.070194	0.313291	9
7	ADHFE1	alcohol dehydrogenase, iron containing, 1	0.046399	0.303681	8
8	ATP4A	ATPase, H+/K+ exchanging, alpha polypeptide	0.172493	0.339041	8
9	CCKBR	cholecystokinin B receptor	0.065433	0.280453	8
10	CPS1	carbamoyl-phosphate synthase 1, mitochondrial	0.083989	0.321429	8
11	HDC	histidine decarboxylase	0.074607	0.304615	8

**Table 2:** Biochemical pathways and their associated genes among 100 nodes of main connected component. The pathways are retrieved from KEGG_20.11.2017. * Corrected with Bonferroni step down, %G/T; percentage of genes per term, and G/T; genes per term

R	GOTerm	P Value	Group	% G/T	G/T	Associated Genes Found
1	Arginine and proline metabolism	0.04	1	6.00	3	[AGMAT, CKB, CKMT2]
2	Mitophagy	0.03	2	4.62	3	[BNIP3, GABARAPL1, MITF]
3	Gastric acid secretion	0.00	3	13.33	10	[ATP4A, ATP4B, CCKBR, CFTR, HRH2, KCNE2, KCNJ16, KCNQ1, SLC26A7, SLC4A2]
4	Arginine biosynthesis	0.04	4	14.29	3	[CPS1, GLUL, GPT2]
5	Alanine, aspartate and glutamate metabolism	0.04	4	8.57	3	[CPS1, GLUL, GPT2]
6	Valine, leucine and isoleucine degradation	0.00	5	10.42	5	[ACAT1, ALDH6A1, BCKDHB, DBT, OXCT1]
7	Propanoate metabolism	0.00	5	12.50	4	[ACAT1, ALDH6A1, BCKDHB, DBT]
8	Pancreatic secretion	0.03	6	4.17	4	[CCKAR, CFTR, KCNQ1, SLC4A2]
9	Bile secretion	0.03	6	4.23	3	[AQP4, CFTR, SLC4A2]

Numbers of 95 isolated nodes were identified which can be included in the network if relevant genes be added to them. In this case numbers of connections also will increase. Due to study interactions between queries, no additional neighbor genes were considered. Degree, betweenness centrality, and closeness centrality are three important central parameters which indicate to imbalance distribution of frequency of interactions between various nodes, non-homogenous controlling of elements of network by a certain node, and unequal propagation rate of information among nodes of different ways. To determine critical nodes which differentiate BE samples from gastric cardia, these three central parameters were considered. Seven crucial nodes including KIT, CFTR, IMPDH2, MYB, FLT1, ATP4A, and CPS1 that are tabulated in table 1 were identified. It is predictable that the critical nodes play significant roles in the integrity and function of the network. As it is depicted in the figure 3, KIT, CFTR, IMPDH2, MYB, and FLT1 (71% of central nodes and 5% of all interacted nodes) are presented in the action map. Only 14% of the non-central nodes or 12% of all interacted nodes are involved in the action map. Gharahkhani *et al*. reported eight new risk loci near CFTR gene in BE patients (21). Up-regulation of c-MYB in BE is reported by Brabender *et al.* however, here v-MYB is down-regulated (22).Therefore, the critical nodes plays more significant role in action map relative to the other DEG. Action map contains three clusters; a paired cluster, a triple ones, and a connected component including 12 nodes. Surprisingly, KIT, CFTR, IMPDH2, MYB, and FLT1are presented in this cluster. It can be concluded that 71% central nodes of network acts as a condensed unit in the network. Except two inhibition actions between CFTR - IMPDH2 and MYBL2 – TFAP2A the other 13 directional connections are activators. There are two up-regulation actions between MYB → KIT and NR2F2 → FLT1 which are formed by central nodes. In figure 4 and table 2, 9 biochemical pathways related to the 100 elements of the main connected component are grouped in 6 clusters and presented in details. Three groups including groups 1, 4, and 5 including 5 pathways are metabolic pathways related to the metabolism of amino acids. Davis *et al.* reported that urinary levels of several amino acid such as alanine, leucine, valine, and glutamine are changed significantly in BE patients (23). These reported data are corresponded to our finding. Term and group 3 are related to gastric acid secretion. As it is reported activation of gastric H^+^/K^+^-ATPase is the final common pathway of acid secretion (24). Since cardia is in contact with gastric acid but esophagus is not, it is a logical finding to differentiate term. Numbers of 10 DEGs (10% of network nodes) are participated in the gastric acid secretion pathways (see table 2). Three central nodes including ATP4A, CPS1, and CFTR (43% of central nodes) are involved in the biochemical pathways. Distribution of DEGs as a function of logarithm of fold change is shown in the figure 5. Numbers of 65 DEGs are down-regulated while 166 ones are up-regulated. DEGs with highest value of fold changes (down-regulated a group and up-regulate f and g groups) are presented in the table 3. Except ATP4A the other central nodes are absent in this table. It can be concluded that expression change of the central nodes are not grossly altered. This finding consist with finding in the figure 3. As showed in action map, prominent action was activation. It seems KIT, CFTR, IMPDH2, MYB, and FLT1 are critical genes which can be considered as drug target in BE patients. Metabolites, especially several amino acids such as alanine, valine, and leucine can be monitored as urinary or probably plasma diagnostic biomarkers in patients (25-27).

Seven central DEGs including KIT, CFTR, IMPDH2, MYB, FLT1, ATP4A, and CPS1 were identified as prominent genes related to BE patients. Further analysis revealed KIT, CFTR, IMPDH2, MYB, and FLT1 play crucial roles relative to ATP4A and CPS1. Role of amino acid metabolism such as arginine, alanine, aspartate, glutamate, valine, leucine and isoleucine in this disorder was highlighted.
